# Dataset of seminal plasma proteome of Nellore bulls with high and low percentage of abnormalities sperm

**DOI:** 10.1186/s13104-024-06796-7

**Published:** 2024-05-10

**Authors:** Camilo Jose Ramirez-Lopez, Edvaldo Barros, Pedro Marcus Vidigal, Lidiany Lopes Gomes, Maria Cristina Baracat-Pereira, Simone Eliza Facioni Guimarães, José Domingos Guimarães

**Affiliations:** 1 Laboratory of Animal Reproduction, Department of Veterinary Medicine, Universidade Federal de Viçosa, Viçosa, Minas Gerais 3657-9000 Brazil; 2 Núcleo de Análise de Biomoléculas, Universidade Federal de Viçosa, Viçosa, Minas Gerais 3657-9000 Brazil; 3 Laboratory of Proteomics and Protein Biochemistry, Department of Biochemistry and Molecular Biology, Universidade Federal de Viçosa, Viçosa, Minas Gerais 3657-9000 Brazil; 4 Laboratory of Animal Biotechnology, Department of Animal Science, Universidade Federal de Viçosa, Viçosa, Minas Gerais 3657-9000 Brazil

**Keywords:** Proteomic, Mass spectrometry, Bos indicus, Bovine, Sire, Semen

## Abstract

**Objectives:**

Bovine seminal plasma proteins perform several functions related to sperm function. Changes in the expression pattern or abundance of seminal proteins are related to changes in the fertilizing capacity of bulls. Considering the role of seminal plasma proteins in sperm function and animal reproduction, we investigated changes in the protein abundance profile in response to sperm morphological changes using a proteomic approach.

**Datadescription:**

In our present investigation, we employed liquid chromatography coupled with mass spectrometry to elucidate the proteomic composition of seminal plasma obtained from Nellore bulls exhibiting varying percentages of sperm abnormalities. Following semen collection, seminal plasma was promptly isolated from sperm, and proteins were subsequently precipitated, enzymatically digested using porcine trypsin, and subjected to analysis utilizing the Acquity nano UHPLC System in conjunction with a mass spectrometer. This dataset encompasses a total of 297 proteins, marking the inaugural instance in which a comparative profile of seminal plasma proteins in young Nellore bulls, categorized by their sperm abnormality percentages, has been delineated using LC-MS/MS. The comprehensive nature of this dataset contributes pivotal proteomic insights, representing a noteworthy advancement in our understanding of the reproductive biology of the Nellore breed.

## Objective

The Breeding Soundness Evaluation is an essential procedure designed to assess factors impacting the reproductive function of males. This involves correlating clinical aspects with the examination of genital organs, semen physical traits, and sperm morphology [1]. However, parameters such as sperm motility and vigor show limited correlation with the in vivo fertility index, possibly due to molecular sperm alterations not perceptible through conventional analyses [2].

The fertilizing capacity of spermatozoa depends on intrinsic cell factors, such as chromatin integrity and sperm morphology, as well as extrinsic factors related to the biochemical composition of seminal plasma [3]. Seminal plasma is the liquid fraction of semen produced by the secretions of the testicles, epididymis, and accessory sexual glands, rich in a variety of proteins that play essential roles in metabolism and modulating the spermatozoa response to different environments and stimuli [4].

Seminal plasma proteins also participate in remodeling the plasma membrane during sperm capacitation and acrosome reaction, playing a crucial role in binding sperm to the zona pellucida of the oocyte and early embryonic development [5]. Changes in the abundance profile of seminal plasma proteins are associated with alterations in the sperm parameters of bulls, making them the focus of various studies aiming to identify potential biomarkers of fertility [6].

Therefore, this article presents experimental data describing the proteomic profile of seminal plasma from Nellore bulls with high and low percentages of sperm abnormalities. This dataset contains valuable information that can enhance our understanding of how seminal plasma proteins respond to anatomical changes in sperm and can support other research in the field of animal reproduction. It can also be used to compare new findings about the proteomic profile of seminal plasma from other cattle breeds and other species.

## Data description

A dataset of 297 seminal plasma proteins from young Nellore bulls with high and low percentages of sperm abnormalities obtained by liquid chromatography-tandem mass spectrometry analysis is provided. We used 20 bulls with an average age of 21.7 months, divided equally into two groups: Low sperm abnormalities (n = 10) and High sperm abnormalities (n = 10). The bulls come from Agropecuaria CFM, a Nellore sire production farm in the city of Aquidauana, Brazil (19° 48’ 53.3124’’ S, 55° 40’ 8.2704’’ W), where animals are raised in the field, fed with Brachiaria brizantha grass and water ad libitum. The bulls were subjected to Breeding Soundness Evaluation on-site at the farm, under individual containment in the appropriate trunk, and semen was collected using the electroejaculation method at the time of evaluation. After collection, the bulls were released [7]. All phenotypic characteristics of the semen were evaluated by optical microscopy and the percentage of sperm abnormalities was calculated by counting 400 cells using phase contrast microscopy (BX41, Olympus, Tokyo, Japan), and the percentage of sperm defects in the acrosome, head, midpiece, and tail was determined [8–9]. Immediately after collection, the ejaculates were centrifuged to separate the seminal plasma from the sperm. Seminal plasma proteins were precipitated using an ice acetone solution and quantified using the Bradford method. For this, an analytical calibration curve was constructed using standard solutions of bovine serum albumin with known concentrations. After incubating the solutions for 10 min at room temperature and darkness, absorbance readings were taken at 595 nm using a spectrophotometer. Triplicate blank readings were also performed using distilled water. Average absorbance readings were calculated, and the readings from the blanks and empty plates were subtracted to obtain a linear equation correlating absorbance with concentration. The protein concentration in the samples was estimated based on the linear relationship established by the calibration curve [10]. . Then, aliquots of 60 µg of protein from each bill sample were added to a new tube to pool two groups of animals. Subsequently, five replicates of each pool containing 50 µg of proteins were subjected to enzymatic digestion in gel, and the generated peptides were mass spectrometry using a nano-Acquity UHPLC system (Waters, Milford, MA, USA) in tandem with a MAXIS 3G model mass spectrometer (Bruker Daltonics, Billerica, MA, USA), operating online with a CaptiveSpray ionization source. Peptide analysis was done by an appropriate method (IE_GCF_01-02-2017). Seminal plasma proteins were identified using MASCOT Daemon v.2.4.0 software by comparing them to a reference database consisting of canonical protein sequences from the Bovidae family (Taxonomy ID 9895), which contained 91,453 entries. This database was accessed via the UniProtKB database (https://www.uniprot.org/taxonomy/9895) on 28 March 2018.The search parameters used for peptide identification were the enzymatic digestion by trypsin with one missed cleavage, cysteine carbamidomethylation as a fixed modification, and methionine oxidation as a variable modification. The error tolerance allowed for the acquired data was 30 ppm for the parental ion and 0.6 DA for the fragments, with the ion charge varying between + 2 and + 4. The validation of Mascot Daemon results was performed using Scaffold Q + version 4.0 (Proteome Software Inc., Portland, OR, USA). Peptide validation was carried out employing the Peptide Prophet algorithm [11, 28], while protein identification was validated using the Protein Prophet algorithm [12, 29]. Validation criteria included a probability threshold of 0.95 for identification, a FDR of ≤ 1%, and a minimum requirement of 2 peptides for protein identification. Furthermore, only proteins identified in a minimum of two replicates were considered for analysis. Protein gene ontology annotations were described. The dataset supporting this article is available in the JPOST REPOSITORY and consists of ten raw files (.zip) and ten processed data files (.mgf) corresponding to the five technical replicates of each animal group (high percentages: INAPTOS; low percentages: APTOS). Additionally, the dataset also includes two additional information files. The first file displays information about identified proteins, including peptide sequence, molecular weight, isoelectric point, identification probability for each peptide, and the percentage of protein identification. On the other hand, the second additional file contains gene ontology annotations for the proteins, including name, gene, biological process, cellular components, and molecular function [13]. The details of the datasets linked to this article are given in Table 1.

**Table 1:** Raw dataset of seminal plasma proteomic of Nellore bulls

Label	Name of data file/data set	File types (file extension)	Data repository and identifier (DOI or accession number)
*Data set 1*	*26,412-POOL2 R2 INAPTOS_1-C,2_01_4917.d*	*Raw file (.zip)*	*JPOST Repository (*https://repository.jpostdb.org/entry/JPST002482*)* [13]
*Data set 2*	*26,412-POOL2 R5 INAPTOS_1-C,5_01_4920.d*	*Raw file (.zip)*	*JPOST Repository (*https://repository.jpostdb.org/entry/JPST002482*))* [13]
*Data set 3*	*26,412-POOL2 R4 INAPTOS_1-C,4_01_4919.d*	*Raw file (.zip))*	*JPOST Repository (*https://repository.jpostdb.org/entry/JPST002482*)* [13]
*Data set 4*	*26,412-POOL2 R3 INAPTOS_1-C,3_01_4918.d*	*Raw file (.zip)*	*JPOST Repository (*https://repository.jpostdb.org/entry/JPST002482*)* [13]
*Data set 5*	*26,412-POOL2 R1 INAPTOS_1-C,1_01_4916.d*	*Raw file (.zip)*	*JPOST Repository (*https://repository.jpostdb.org/entry/JPST002482*)* [13]
*Data set 6*	*26,412-POOL1 R4 APTOS_1-B,4_01_4914.d*	*Raw file (.zip)*	*JPOST Repository (*https://repository.jpostdb.org/entry/JPST002482*)* [13]
*Data set 7*	*26,412-POOL1 R5 APTOS_1-B,5_01_4915.d*	*Raw file (.zip)*	*JPOST Repository (*https://repository.jpostdb.org/entry/JPST002482*)* [13]
*Data set 8*	*26,412-POOL1 R3 APTOS_1-B,3_01_4913.d*	*Raw file (.zip)*	*JPOST Repository (*https://repository.jpostdb.org/entry/JPST002482*)* [13]
*Data set 9*	*26,412-POOL1 R2 APTOS_1-B,2_01_4912.d*	*Raw file (.zip)*	*JPOST Repository (*https://repository.jpostdb.org/entry/JPST002482*)* [13]
*Data set 10*	*26,412-POOL1 R1 APTOS_1-B,1_01_4911.d*	*Raw file (.zip)*	*JPOST Repository (*https://repository.jpostdb.org/entry/JPST002482*)* [13]
*Data file 1*	*26,412-POOL1 R3 APTOS_1-B,3_01_4913_uncalib_4.3.110*	*Peak file (.mgf)*	*JPOST Repository (*https://repository.jpostdb.org/entry/JPST002482*)* [13]
*Data file 2*	*26,412-POOL1 R4 APTOS_1-B,4_01_4914_uncalib_4.3.110*	*Peak file (.mgf)*	*JPOST Repository (*https://repository.jpostdb.org/entry/JPST002482*)* [13]
*Data file 3*	*26,412-POOL1 R5 APTOS_1-B,5_01_4915_uncalib_4.3.110*	*Peak file (.mgf)*	*JPOST Repository (*https://repository.jpostdb.org/entry/JPST002482*)* [13]
*Data file 4*	*26,412-POOL1 R2 APTOS_1-B,2_01_4912_uncalib_4.3.110*	*Peak file (.mgf)*	*JPOST Repository (*https://repository.jpostdb.org/entry/JPST002482*)* [13]
*Data file 5*	*26,412-POOL1 R1 APTOS_1-B,1_01_4911_uncalib_4.3.110*	*Peak file (.mgf)*	*JPOST Repository (*https://repository.jpostdb.org/entry/JPST002482*)* [13]
*Data file 6*	*26,412-POOL2 R5 INAPTOS_1-C,5_01_4920_uncalib_4.3.110*	*Peak file (.mgf)*	*JPOST Repository (*https://repository.jpostdb.org/entry/JPST002482*)* [13]
*Data file 7*	*26,412-POOL2 R3 INAPTOS_1-C,3_01_4918_uncalib_4.3.110*	*Peak file (.mgf)*	*JPOST Repository (*https://repository.jpostdb.org/entry/JPST002482*)* [13]
*Data file 8*	*26,412-POOL2 R4 INAPTOS_1-C,4_01_4919_uncalib_4.3.110*	*Peak file (.mgf)*	*JPOST Repository (*https://repository.jpostdb.org/entry/JPST002482*)* [13]
*Data file 9*	*26,412-POOL2 R2 INAPTOS_1-C,2_01_4917_uncalib_4.3.110*	*Peak file (.mgf)*	*JPOST Repository (*https://repository.jpostdb.org/entry/JPST002482*)* [13]
*Data file 10*	*26,412-POOL2 R1 INAPTOS_1-C,1_01_4916_uncalib_4.3.110*	*Peak file (.mgf)*	*JPOST Repository (*https://repository.jpostdb.org/entry/JPST002482*)* [13]
*Additional data file 1*	*Characterization of seminal plasma proteins*	*MS Excel file (.xlsx)*	*JPOST Repository (*https://repository.jpostdb.org/entry/JPST002482*)* [13]
*Additional data file 2*	*Functional classification of seminal plasma proteins*	*MS Excel file (.xlsx)*	*JPOST Repository (*https://repository.jpostdb.org/entry/JPST002482*)* [13]

## Limitations

The seminal plasma samples were collected in a single moment due to technical limitations that prevented new semen collections and evaluation of the sperm quality of the bulls. Furthermore, the data presented correspond to technical replicates of sample pools from each group of bulls, causing the loss of individual variability in the results. Moreover, the data was generated using the nano-LC-MS/MS system and therefore the resolution is slightly lower compared to other high-resolution data acquisition platforms such as Orbitrap.

## Data Availability

The data described in this data note were submitted to the Japan ProteOme STandard Repository, a member of the ProteomeXchange Consortium. They can be freely and openly accessed through the following link: https://repository.jpostdb.org/entry/JPST002482.

## Abbreviations

UHPCL: Ultra-High Performance Liquid Chromatography
DA: Dalton
FDR: False Discovery Rate
